# Metabolic Alterations Associated With Diet and Hypertension in Rats

**DOI:** 10.1155/jnme/6248625

**Published:** 2026-01-02

**Authors:** Kristina Smoradkova, Mateusz Szudzik, Klaudia Maksymiuk, Emilia Samborowska, Adrian Drapala, Marcin Ufnal, Lenka Tomasova

**Affiliations:** ^1^ Institute of Clinical and Translational Research, Biomedical Research Center, Slovak Academy of Sciences, Dubravska Cesta 9, Bratislava, 845 05, Slovakia, sav.sk; ^2^ Department of Experimental Physiology and Pathophysiology, Laboratory of Centre for Preclinical Research, Medical University of Warsaw, Warsaw, 02-091, Poland, wum.edu.pl; ^3^ Mass Spectrometry Laboratory, Institute of Biochemistry and Biophysics, Polish Academy of Sciences, Warsaw, 00-330, Poland, pan.pl

**Keywords:** dietary patterns, hypertension, LC–MS, methylglycines, taurine

## Abstract

Unhealthy diet and hypertension constitute major risk factors for the development of cardiometabolic diseases. However, the direct effects of dietary components and high blood pressure on metabolic profiles remain poorly understood. We evaluated concentrations of amino acids and nutrients in the plasma and tissues of animal models subjected to high‐fat and high‐disaccharide diet without excessive caloric intake, in salt‐resistant rats on high salt intake, in spontaneously hypertensive rats, and in angiotensin II–induced hypertensive rats. Using liquid chromatography and mass spectrometry, we identified changes in several analytes across models. We found that a high‐fat diet increased tissue levels of methylglycines (betaine, sarcosine) and glycine, while high salt intake and hypertension were associated with a distinct reduction of methyl/glycine species and the accumulation of taurine in the tissues. We further observed tissue‐specific alterations. For instance, alanine was decreased in the kidneys of rats on high salt and in hypertensive models. Beta‐alanine was higher in the lung and renal cortex of rats on high salt, but lower in the cardiovascular system of hypertensive models. A high‐sugar diet increased circulating levels of betaine and taurine, although its impact on tissues was less pronounced. In conclusion, this study provides a comprehensive evaluation of organic solutes in different animal models and highlights the diverse metabolic alterations associated with diet and hypertension. Further research is required to explore the significance of these findings and their potential implications for understanding disease mechanisms.

## 1. Introduction

The transformation of dietary patterns in the last few decades represents a global health concern. Excessive intake of saturated fat and simple carbohydrates has been associated with the development of dyslipidemia and diabetes [1]. Hypertension is another leading risk factor for morbidity and mortality. The prevalence of hypertension is high and still tends to rise [2]. The majority of hypertensive cases is idiopathic and belongs to the category of essential hypertension. Various mechanisms were proposed in the literature, including an impaired response of the renin‐aldosterone system, increased activation of the sympathetic nervous system, and renal sodium retention [3]. The latter is associated with exaggerated blood pressure elevation due to excessive salt intake in salt‐sensitive subjects.

Amino acids and nutrients that supply the one‐carbon metabolism are crucial for the maintenance of methylation capacity. Glycine and its methylated derivatives serve as donors of one‐carbon units. Betaine (trimethylglycine) donates a methyl group to the methionine cycle. The reversible demethylation of betaine to sarcosine (methylglycine) and glycine can directly contribute carbon units to the folate cycle. Alternatively, the one‐carbon unit may originate from the reaction that derives glycine from serine, an intermediate product in the synthesis and degradation of alanine. The folate cycle is completed by the methylation of homocysteine and the formation of methionine. In the case that homocysteine does not enter the methionine cycle by one of these pathways, it can be converted in the transsulfuration pathway to taurine and other sulfur derivatives. The methylation processes that are dependent on the availability of one‐carbon donors modify the epigenetic status of the cells, thus modulating the activity of enzymes, gene expression, and receptor sensitivity [4]. For instance, it was shown that methylation is involved in the control of lipid metabolism and blood pressure [5, 6]. Particularly, the methylation levels correlated with the expression of sodium‐coupled transporters, lipid composition, or salt memory phenomenon [7–9].

The current study evaluates the effect of dietary patterns and hypertension on the levels of glycine and other metabolic‐ or structural‐related organic solutes. Namely, we analyzed plasma and tissues of rats on high‐fat and high‐sugar diet without excessive caloric intake, salt‐resistant rats on high salt intake, spontaneously hypertensive rats (SHR), and angiotensin II (Ang II)–induced hypertensive rats by liquid chromatography coupled with mass spectrometry (LC–MS). The grouping was designed to evaluate how different diet‐induced and genetically predisposed conditions, all associated with metabolic and cardiovascular disturbances, affect amino acid metabolism. This approach enables us to identify potential shared or distinct metabolic alterations linked to dietary and hypertensive pathophysiology.

## 2. Material and Methods

### 2.1. Animals

The study was conducted on male Sprague Dawley rats, Wistar Kyoto rats (WKY), and SHRs. Animals were obtained from the Central Laboratory for Experimental Animals, Medical University of Warsaw, Poland, and housed in groups of 2–4 in propylene cages, temperature 22°C–23°C, humidity 45%–55%, and 12 h light/12 h dark cycle. The animal study was conducted in the Laboratory of the Centre for Preclinical Research Laboratory, Medical University of Warsaw. The experiments were performed according to Directive 2010/63 EU on the protection of animals used for scientific purposes and approved by the Local Bioethical Committee (no: WAW2/061/2021, WAW2/082/2018, and WAW2/555/2018).

Three series of rats were analyzed: (i) 12–14‐week‐old Sprague Dawley rats on tap water (*n* = 8), on low salt dose for 2 weeks (0.9% NaCl, *n* = 8), and on high salt dose for 2 weeks (1.5% NaCl, *n* = 8); (ii) 6‐week‐old Sprague Dawley on standard laboratory diet (C 1090–10, Altromin, Lage, Germany; *n* = 8), on disaccharide‐rich diet for 12 weeks (C 1010, Altromin, Lage, Germany; *n* = 7), and on fat‐rich diet for 12 weeks (C 1090–45, Altromin, Lage, Germany; *n* = 7). Diet specification and caloric dose adjustment were performed as described in [10]. (iii) 12‐week‐old WKY (*n* = 6), SHR (*n* = 6), and WKY rats implanted at the age of 10 weeks with a subcutaneous osmotic minipump (*n* = 6). The minipumps (ALZET 2ML; Durect, Cupertino, CA) were releasing Ang II at the rate of (0.76 pmol·s^−1^; 0.8 ng·s^−1^) for 2 weeks as previously described [11]. All surgical procedures were performed using general anesthesia with ketamine/xylazine (100/10 mg/kg of body weight, i.p.) or urethane (1.5 g/kg of body weight, i.p.). At the end of the experiment, plasma and tissue samples were collected immediately and snap frozen at −80°C. The levels of taurine, betaine, sarcosine, glycine, alanine, and beta‐alanine were determined by mass spectrometry coupled with liquid chromatography as we have described previously [12, 13]. These six analytes were selected because (i) they are key components of one‐carbon metabolism, osmolyte regulation, and energy metabolism and (ii) they were consistently detected and quantified across all three experimental series and in all analyzed tissues, allowing direct comparative analysis between groups using a uniform set of metabolites. Briefly, tissues were placed in 10% ethanol and homogenized using the Precellys Cryolys Evolution tissue homogenizer (Bertin Instruments, Montigny‐le‐Bretonneux, France). The concentrations of organic solutes were evaluated using a Waters Acquity Ultra Performance Liquid Chromatograph (Waters, Milford, MA, USA) coupled to a Waters TQ‐S triple‐quadrupole mass spectrometer through an electrospray ionization source. For the instrument control and data acquisition, Waters MassLynx software was used (Waters, Manchester, UK). Waters TargetLynx was used to process data (Waters, Manchester, UK). The concentrations were determined in dry tissue mass. The mass spectrometer was operated in multiple‐reaction monitoring (MRM) mode, with two MRM transitions defined for each analyte. Calibration standards were prepared in water, as amino acid‐free serum or urine matrices were not available. Calibration curves were generated by plotting the ratio of the analyte peak area to that of the corresponding internal standard, which consisted of isotopically labeled analogs of the analytes, against known analyte concentrations. The method demonstrated linearity over the following concentration ranges: L‐alanine 19.08–1908 μmol/L, β‐alanine 1.9–190.8 μmol/L, sarcosine 0.19–47 μmol/L, taurine 6.8–1358 μmol/L, glycine 11.3–566 μmol/L, and betaine 1.7–340 μmol/L. Plasma and tissue sample concentrations were determined by comparison with the obtained calibration curves. The mean coefficients of calibration curves (R2) were not lower than 0.98. The method exhibited good reproducibility, with variability at the level of approximately 10%.

### 2.2. Statistics

Outliers were defined by the ROUT method (*Q* = 0.9%) and removed from the statistical analysis. The Shapiro–Wilk test was used to test the normality of the distribution. Differences were evaluated by the Kruskal–Wallis test followed by post hoc Dunn’s test. A value of two‐sided *p* < 0.05 was considered significant. Statistical analysis was conducted using GraphPad Prism 8.0.2 (GraphPad Software, Inc.; San Diego, USA).

## 3. Results

### 3.1. The Influence of High Salt Intake on the Levels of Organic Solutes in the Plasma and Tissues of Rats

The intake of 0.9% and 1.5% salt solution over 2 weeks significantly altered the concentrations of organic solutes in the plasma, heart, lung, liver, and kidney of rats without influencing the blood pressure of rats. Changes of organic solutes in the plasma and tissues of rats drinking salt solution are shown in Table 1. Plasmatic levels of sarcosine were lower in the group of rats drinking 0.9% NaCl in comparison with the control group (2‐fold, *p* < 0.05). The cardiac levels of sarcosine were lower in the group of rats drinking 1.5% NaCl than in the control group (1.4‐fold, *p* < 0.01). Beta‐alanine was higher in the lungs of rats drinking 0.9% NaCl (1.4‐fold, *p* < 0.05) and 1.5% NaCl (1.5‐fold, *p* < 0.01) in comparison with the control group. The concentration of taurine in the lung was higher in the group of rats drinking 1.5% NaCl than in the control group (1.1‐fold, *p* < 0.05). Alanine and glycine levels were lower in the liver of rats drinking 0.9% NaCl (1.2‐fold, *p* < 0.05) and 1.5% NaCl (1.2‐fold, *p* < 0.01), respectively, than in the control group. We have detected higher levels of beta‐alanine in the renal cortex of rats drinking 1.5% NaCl than in the control group (1.7‐fold, *p* < 0.01). The concentrations of sarcosine (1.4‐fold, *p* < 0.05) and alanine (1.1‐fold, *p* < 0.05) were lower in the renal medulla of rats drinking 0.9% NaCl than in the control group. Decreased were the concentrations of glycine in the renal medulla of rats drinking 1.5% NaCl in comparison with rats drinking 0.9% NaCl (1.1‐fold, *p* < 0.05).

**Table 1:** Levels of organic solutes in the plasma and tissues of rats on high salt.

	Control	0.9% NaCl	1.5% NaCl	Kruskal–Wallis test
Plasma (μmol/L)				
Betaine	184.6 (113; 312.7)	134.6 (102.8; 211.0)	164.1 (134.3; 195.2)	*p* = 0.658
Glycine	146.3 (139.7; 152)	155.5 (144.5; 170.9)	147.8 (130.2; 171.1)	*p* = 0.508
Sarcosine	3.55 (2.13; 4.95)	**2.01 (1.933; 2.62)** ^**∗**^	2.34 (1.993; 2.915)	**p** = 0.039
Alanine	243.7 (226.1; 286.7)	224.3 (181.3; 286.1)	219.5 (197.6; 303.8)	*p* = 0.585
Beta‐alanine	2.5 (1.44; 3.76)	4.0 (2.73; 6.94)	3.585 (2.835; 6.325)	*p* = 0.190
Taurine	836.4 (806.7; 1061)	588.1 (375.3; 738.9)	572.1 (430.5; 1097)	*p* = 0.087
Heart (μmol/kg)				
Betaine	267.6 (198.5; 310.7)	263.3 (246.9; 309.4)	260.2 (244.8; 270.1)	*p* = 0.988
Glycine	1131 (1085; 1150)	1127 (1087; 1268)	1129 (1049; 1174)	*p* = 0.940
Sarcosine	6.74 (5.50; 8.69)	5.81 (5.59; 6.19)	**4.96 (4.73; 5.44)** ^**∗∗**^	**p** = 0.003
Alanine	3031 (2968; 3213)	2929 (2877; 3209)	3210 (3119; 3426)	*p* = 0.258
Beta‐alanine	57.06 (54.30; 62.48)	75.03 (48.45; 76.85)	62.88 (56.84; 68.59)	*p* = 0.150
Taurine	36,441 (35,129; 38,374)	36,734 (34,790; 38,760)	37,896 (36,480; 39,718)	*p* = 0.359
Lung (μmol/kg)				
Betaine	287.5 (271.9; 401.4)	326.6 (296.7; 446.3)	382.5 (331.6; 390.3)	*p* = 0.190
Glycine	8005 (7331; 8312)	8234 (7897; 8468)	8014 (7897; 8600)	*p* = 0.398
Sarcosine	25.72 (17.54; 31.89)	19.50 (16.75; 23.56)	21.20 (18.15; 22.47)	*p* = 0.408
Alanine	4646 (4233; 4947)	4405 (4050; 5383)	4404 (3952; 4954)	*p* = 0.684
Beta‐alanine	54.96 (44.11; 67.14)	**77.26 (57.48; 93.49)** ^**∗**^	**84.27 (77.62; 90.21)** ^**∗∗**^	**p** = 0.001
Taurine	10,016 (9428; 10,419)	10,070 (10,006; 12,130)	**11,357 (10,914; 11,798)** ^**∗**^	**p** = 0.014
Liver (μmol/kg)				
Betaine	2282 (1922; 2959)	2897 (2512; 4412)	3029 (2335; 3688)	*p* = 0.268
Glycine	4192 (3908; 4321)	3825 (3695; 4482)	**3532 (3375; 3808)** ^**∗∗**^	**p** = 0.011
Sarcosine	121.8 (82.42; 143.3)	61.74 (48.38; 107.4)	73.71 (69.96; 91.98)	*p* = 0.059
Alanine	16,058 (15,400; 17,807)	**13,194 (12,024; 13,832)** ^**∗**^	14,738 (13,559; 15,160)	**p** = 0.021
Beta‐alanine	399.1 (382.5; 446.9)	421.7 (383.8; 490)	402.2 (372.7; 446.5)	*p* = 0.644
Taurine	6324 (5353; 7821)	4893 (3248; 5174)	5173 (4170; 7027)	*p* = 0.079
Renal cortex (μmol/kg)				
Betaine	1616 (1247; 2327)	2179 (1467; 2236)	1540 (1395; 1848)	*p* = 0.521
Glycine	13,739 (13,358; 14,294)	14,501 (14,019; 15,493)	13,276 (12,621; 14,407)	*p* = 0.122
Sarcosine	7.57 (6.26; 10.94)	5.57 (5.34; 7.22)	6.44 (5.73; 6.76)	*p* = 0.106
Alanine	13,355 (12,329; 14,003)	13,317 (12,714; 14,648)	13,288 (12,256; 14,319)	*p* = 0.834
Beta‐alanine	56.02 (53.34; 73.27)	76.16 (57.13; 99.48)	**97.14 (76.33; 124.9)** ^**∗∗**^	**p** = 0.008
Taurine	13,438 (12,760; 15,499)	11,609 (10,525; 12,998)	11,844 (10,550; 16,099)	*p* = 0.094
Renal medulla (μmol/kg)				
Betaine	1554 (1465; 1685)	2111 (1460; 2502)	2020 (1926; 2832)	*p* = 0.079
Glycine	13,073 (12,038; 13,771)	14,344 (13,275; 15,327)	**12,325 (11,584; 13,322)** ^#^	**p** = 0.018
Sarcosine	10.19 (8.02; 11.97)	**7.41 (6.55; 8.64)** ^**∗**^	8.14 (7.1; 9.11)	**p** = 0.034
Alanine	13,855 (12,588; 14,468)	**12,681 (12,418; 14,402)** ^**∗**^	11,783 (10,312; 12,603)	**p** = 0.043
Beta‐alanine	77 (68.46; 89.26)	96.49 (76.20; 127.2)	93.68 (79.80; 134.6)	*p* = 0.074
Taurine	12,414 (10,967; 13,279)	9189 (8626; 11,750)	10,659 (7585; 13,624)	*p* = 0.088

*Note:* All data are expressed as the median (Q1; Q3) Kruskal–Wallis test followed by post hoc Dunn’s test. Bold values represent *p* values that are statistically significant.

^∗^ *p* < 0.05 vs. control.

^∗∗^ *p* < 0.01 vs. control.

^#^ *p* < 0.05 vs. 0.9% NaCl.

### 3.2. The Influence of High Sugar and High‐Fat Diet on the Levels of Organic Solutes in the Plasma and Tissues of Rats

The intake of a high‐disaccharide diet with caloric restriction for 12 weeks significantly altered the concentrations of organic solutes in the plasma and heart. The intake of high‐fat diet with caloric restriction for 12 weeks significantly altered the concentrations of organic solutes in the plasma, heart, lung, liver, and kidney of rats. Changes of organic solutes in the plasma and tissues of rats on high‐fat and high‐sugar diet are shown in Table 2. Rats on high‐sugar diet showed higher plasma levels of betaine (1.4‐fold, *p* < 0.01) and taurine (1.6‐fold, *p* < 0.05) in comparison with the control group. Plasmatic glycine levels were higher in the group of rats on high‐fat diet in comparison with the control group (3.6‐fold, *p* < 0.01) and the high‐sugar diet group (3.5‐fold, *p* < 0.01). Cardiac levels of betaine were higher in rats on high‐sugar diet in comparison with the control group (1.5‐fold, *p* < 0.05). Rats on high‐sugar diet showed lower levels of glycine in the heart in comparison with the control group (1.4‐fold, *p* < 0.05) and the high‐fat diet group (1.7‐fold, *p* < 0.05). The levels of sarcosine in the heart of rats on high‐fat diet were lower in comparison with the control group (1.7‐fold, *p* < 0.05) and the high‐sugar diet group (1.5‐fold, *p* < 0.05). Cardiac levels of taurine were higher in the high‐fat group in comparison with the control group (1.2‐fold, *p* < 0.05). High‐fat diet decreased the levels of taurine in the lungs (1.2‐fold, *p* < 0.01) and liver (1.7‐fold, *p* < 0.01) in comparison with the control group. We detected higher levels of hepatic betaine in the group of rats on high‐fat diet than in the control group (1.6‐fold, *p* < 0.01) and in the high‐sugar diet group of rats (1.6‐fold, *p* < 0.05). Hepatic glycine levels in rats on high‐fat diet were increased in comparison with the control group (1.4‐fold, *p* < 0.05) and the group of rats on high‐sugar diet (1.5‐fold, *p* < 0.001). Taurine levels in the liver were lower in the group of rats on high‐fat diet in comparison with the control group (1.7‐fold, *p* < 0.01). High‐fat diet increased the levels of glycine in the renal cortex of rats in comparison with the control group (1.2‐fold, *p* < 0.01) and the high‐sugar diet group (1.2‐fold, *p* < 0.05), and in the renal medulla in comparison with the control group (1.1‐fold, *p* < 0.05). Sarcosine levels in the renal cortex of the high‐fat diet group were higher than the control group in renal cortex (1.7‐fold, *p* < 0.01) and renal medulla (1.2‐fold, *p* < 0.05).

**Table 2:** Levels of organic solutes in the plasma and tissues of rats on high‐saccharide and high‐fat diet.

	Control	High‐sugar diet	High‐fat diet	Kruskal–Wallis test
Plasma (μmol/L)				
Betaine	39.48 (34.88; 45.75)	**53.50 (48.10; 60.87)** ^**∗∗**^	45.18 (36.11; 49.13)	**p** = 0.003
Glycine	96.28 (88.06; 109.0)	99.24 (83.73; 102.4)	**342.7 (320.7; 363.7)** ^**∗∗##**^	**p** = 0.0001
Sarcosine	5.240 (4.298; 5.968)	5.290 (4.440; 5.530)	5.670 (5.460; 7.160)	*p* = 0.155
Alanine	374.5 (341.3; 464.7)	393.3 (347.9; 462.1)	289.0 (259.0; 374.9)	*p* = 0.055
Beta‐alanine	4.010 (3.610; 4.800)	5.110 (3.060; 5.440)	3.290 (2.690; 3.400)	**p** = 0.021
Taurine	140.4 (123.7; 169.8)	**227.2 (194.1; 264.1)** ^**∗**^	168.2 (141.2; 192.8)	**p** = 0.021
Heart (μmol/kg)				
Betaine	50.71 (45.66; 61.63)	**75.02 (63.22; 80.26)** ^**∗**^	60.95 (51.45; 61.85)	**p** = 0.018
Glycine	898.2 (731.8; 1180)	**621.9 (616.9; 686.7)** ^**∗**^	**1079 (940.6; 1143)** ^**#**^	**p** = 0.004
Sarcosine	1.130 (0.9225; 1.205)	1.100 (0.870; 1.580)	**1.910 (1.470; 2.14)** ^**∗#**^	**p** = 0.012
Alanine	2825 (2545; 3068)	2682 (2540; 3037)	2674 (2340; 3019)	*p* = 0.769
Beta‐alanine	124.4 (112.5; 137.5)	112.9 (104.7; 143.5)	90.69 (50.46; 114.3)	*p* = 0.083
Taurine	28,089 (26,099; 29,944)	32,989 (30,395; 35,775)	**32,934 (32,182; 36,323)** ^**∗**^	**p** = 0.018
Lung (μmol/kg)				
Betaine	138.5 (125.2; 142.7)	161.9 (151.9; 191.7)	127.7 (117.3; 153.4)	*p* = 0.037
Glycine	6320 (6021; 6683)	6504 (6436; 6618)	6779 (5202; 7295)	*p* = 0.424
Sarcosine	7.460 (6.455; 8.768)	7.930 (7.450; 8.950)	7.600 (7.280; 8.410)	*p* = 0.618
Alanine	4356 (3961; 4802)	4761 (3839; 4840)	4043 (3559; 4772)	*p* = 0.581
Beta‐alanine	94.85 (82.91; 114.3)	87.77 (79.40; 112.3)	88.54 (61.52; 92.46)	*p* = 0.366
Taurine	10,010 (8884; 10,628)	9588 (8500; 9966)	**8402 (6431; 8640)** ^**∗∗**^	**p** = 0.005
Liver (μmol/kg)				
Betaine	687.4 (573.7; 776.6)	712.6 (641.6; 774.3)	**1133 (900.8; 1337)** ^**∗∗#**^	**p** = 0.001
Glycine	3573 (3555; 3595)	3233 (2858; 3438)	**4864 (3922; 5230)** ^**∗###**^	**p** < 0.0001
Sarcosine	30.48 (19.44; 37.25)	22.09 (15.50; 30.50)	33.34 (32.31; 39.37)	*p* = 0.117
Alanine	12,575 (11,767; 13,445)	11,622 (10,497; 12,863)	10,981 (10,474; 11,896)	*p* = 0.051
Beta‐alanine	336.0 (297.2; 372.0)	412.7 (296.2; 420.9)	305.4 (273.3; 320.7)	*p* = 0.273
Taurine	8978 (7353; 9445)	7797 (6813; 8371)	**5308 (4557; 5816)** ^**∗∗**^	*p* = 0.002
Renal cortex (μmol/kg)				
Betaine	1391 (1070; 1532)	1415 (1191; 1541)	1332 (1152; 1554)	*p* = 0.8891
Glycine	6012 (4688; 6224)	5789 (5245; 6535)	**6996 (6896; 7311)** ^**∗∗##**^	**p** < 0.0001
Sarcosine	4.745 (3.975; 5.675)	6.100 (5.550; 7.030)	**7.920 (5.740; 8.270)** ^**∗∗**^	**p** = 0.0029
Alanine	11,358 (9189; 11,699)	12,447 (11,858; 13,991)	11,230 (10,641; 11,748)	**p** = 0.0491
Beta‐alanine	95.19 (86.42; 156.4)	116.2 (102.1; 135.1)	106.1 (90.73; 115.6)	*p* = 0.5729
Taurine	8590 (7487; 10,087)	10,795 (8235; 10,975)	8071 (6752; 9752)	*p* = 0.347
Renal medulla (μmol/kg)				
Betaine	1500 (1397; 1722)	1533 (1148; 1941)	1311 (847.7; 1556)	*p* = 0.298
Glycine	6058 (5369; 6293)	6094 (5718; 6445)	**6703 (6404; 6857)** ^**∗**^	**p** = 0.026
Sarcosine	4.300 (2.913; 4.775)	4.100 (3.730; 5.210)	**5.240 (4.860; 6.020)** ^**∗**^	**p** = 0.013
Alanine	12,886 (10,876; 13,412)	12,179 (10,839; 12,954)	10,762 (10,218; 12,158)	*p* = 0.070
Beta‐alanine	87.39 (84.20; 94.93)	93.66 (77.40; 112.4)	79.44 (75.26; 115.5)	*p* = 0.870
Taurine	9328 (8119; 10,795)	9767 (7947; 11,904)	7322 (6945; 9050)	*p* = 0.057

*Note:* All data are expressed as the median (Q1; Q3) Kruskal–Wallis test followed by post hoc Dunn’s test. Bold values represent *p* values that are statistically significant.

^∗^ *p* < 0.05 vs. control.

^∗∗^ *p* < 0.01 vs. control.

^#^ *p* < 0.05 vs. high‐sugar diet.

^##^ *p* < 0.01 vs. high‐sugar diet.

^###^ *p* < 0.001 vs. high‐sugar diet.

### 3.3. The Influence of High Blood Pressure on the Levels of Organic Solutes in the Plasma and Tissues of Rats

SHRs showed altered concentrations of organic solutes in the plasma, heart, lung, liver, and kidney. Rats with Ang II–induced hypertension showed altered levels of organic solutes in the plasma, heart, lung, and kidney. Changes of organic solutes in the plasma and tissues of rats on high‐fat and high‐sugar diet are shown in Table 3. Ang II hypertensive group showed significantly lower levels of glycine compared to normotensive group (1.3‐fold, *p* < 0.05) and spontaneously hypertensive group (1.4‐fold, *p* < 0.05). Plasmatic levels of sarcosine (1.7‐fold, *p* < 0.05) and beta‐alanine (7.7‐fold, *p* < 0.01) were significantly lower in spontaneously hypertensive group compared to normotensive group. We detected lower levels of plasmatic taurine in Ang II hypertensive group in comparison with spontaneously hypertensive group (2.2‐fold, *p* < 0.05). Cardiac sarcosine levels were lower spontaneously hypertensive group compared to normotensive group (1.9‐fold, *p* < 0.05). Cardiac beta‐alanine levels were significantly lower in Ang II hypertensive group compared to normotensive group (1.6‐fold, *p* < 0.01). Betaine levels in the lungs of spontaneously hypertensive group were significantly lower than normotensive group (1.9‐fold, *p* < 0.05) and Ang II hypertensive group (2‐fold, *p* < 0.05). Similarly, sarcosine levels in the lungs of spontaneously hypertensive group were lower compared to normotensive group (1.6‐fold, *p* < 0.01) and Ang II hypertensive group (1.5‐fold, *p* < 0.05). Hepatic betaine was lower in spontaneously hypertensive group compared to Ang II hypertensive group (1.8‐fold, *p* < 0.05). Hepatic levels of glycine (1.3‐fold, *p* < 0.05) and alanine (1.2‐fold, *p* < 0.01) were significantly lower in the Ang II hypertensive group compared to spontaneously hypertensive group. Levels of taurine in the liver of spontaneously hypertensive group were significantly higher than in the normotensive group (1.8‐fold, *p* < 0.05) and Ang II hypertensive group (2‐fold, *p* < 0.05). We detected lower levels of glycine in the renal cortex of Ang II hypertensive group (1.2‐fold, *p* < 0.01) in comparison with normotensive group. Sarcosine levels in spontaneously hypertensive group were significantly lower in renal cortex (1.8‐fold, *p* < 0.01, and 1.9‐fold, *p* < 0.05) and renal medulla (1.4‐fold, *p* < 0.05, and 1.6‐fold, *p* < 0.01) compared to the normotensive group and Ang II hypertensive group, respectively. The levels of alanine in the renal cortex were significantly lower in spontaneously hypertensive group (1.3‐fold, *p* < 0.05) and Ang II hypertensive group (1.4‐fold, *p* < 0.01) in comparison with the control group. The spontaneously hypertensive group of rats had higher levels of taurine in renal cortex in comparison with normotensive group (1.9‐fold, *p* < 0.05) and Ang II hypertensive group (2.4‐fold, *p* < 0.01). Alanine levels in renal medulla were lower in Ang II hypertensive group in comparison with the normotensive group (1.3‐fold, *p* < 0.001). Ang II hypertensive group showed significantly lower levels of taurine in renal medulla (2.3‐fold, *p* < 0.05) in compared to spontaneously hypertensive group.

**Table 3:** Levels of organic solutes in the plasma and tissues of hypertensive rats.

	Normotensive	Spontaneously hypertensive	Ang II hypertensive	Kruskal–Wallis test
Plasma (μmol/L)				
Betaine	293.3 (133.6; 432.3)	106.8 (95.77; 184.9)	260.2 (222.3; 329.2)	*p* = 0.0229
Glycine	168.2 (160.4; 241.9)	173.3 (158.0; 192.8)	**125.2 (105.3; 148.9)** ^**∗#**^	**p** = 0.0021
Sarcosine	3.042 (2.121; 6.114)	**1.805 (1.612; 2.398)** ^**∗**^	2.513 (1.986; 2.904)	**p** = 0.0421
Alanine	178.2 (159.1; 257.2)	235.6 (187.0; 254.9)	159.1 (123.2; 204.0)	*p* = 0.0916
Beta‐alanine	50.65 (41.35; 82.96)	**6.604 (4.008; 8.525)** ^**∗∗**^	40.56 (33.91; 48.88)	**p** < 0.0001
Taurine	378.4 (220.7; 827.0)	993.0 (643.6; 1046)	**453.9 (227.3; 552.3)** ^**#**^	*p* = 0.0231
Heart (μmol/kg)				
Betaine	397.6 (346.7; 456.7)	379.1 (256.6; 488.1)	433.7 (367.5; 562.7)	*p* = 0.5367
Glycine	1524 (1450; 1648)	1517 (1369; 1725)	1382 (1292; 1574)	*p* = 0.2475
Sarcosine	3.140 (2.352; 4.376)	**1.639 (1.552; 2.145)** ^**∗**^	2.516 (2.283; 3.087)	**p** = 0.0059
Alanine	3439 (3315; 3631)	3248 (2998; 3492)	3347 (3130; 3497)	*p* = 0.2761
Beta‐alanine	96.17 (89.29; 107.7)	89.16 (64.66; 96.47)	**60.44 (53.49; 77.09)** ^**∗∗**^	**p** = 0.0033
Taurine	26,062 (24,884; 28,455)	27,031 (25,211; 27,540)	27,958 (26,194; 29,033)	*p* = 0.6226
Lung (μmol/kg)				
Betaine	605.5 (473.1; 757.6)	**322.2 (283.7; 447.8)** ^**∗**^	**654.9 (535.6; 679.8)** ^**#**^	**p** = 0.0089
Glycine	10,100 (9148; 10,855)	10,076 (9505; 11,282)	9442 (8880; 10,297)	*p* = 0.4838
Sarcosine	19.68 (16.13; 31.02)	**12.38 (8.635; 13.86)** ^**∗∗**^	**18.46 (14.87; 21.41)** ^**#**^	**p** = 0.0007
Alanine	4774 (4567; 5384)	6127 (5289; 6892)	5324 (4508; 5893)	*p* = 0.082
Beta‐alanine	64.94 (54.64; 77.89)	56.33 (42.36; 61.25)	55.16 (42.77; 68.06)	*p* = 0.1962
Taurine	9527 (9207; 10,349)	8789 (7673; 9608)	10,070 (8473; 11,022)	*p* = 0.2171
Liver (μmol/kg)				
Betaine	2897 (2795; 3046)	1684 (1333; 2196)	**3079 (2526; 5248)** ^**#**^	**p** = 0.0047
Glycine	7234 (6887; 7507)	8175 (7649; 8706)	**6476 (6216;** **7469)** ^**#**^	**p** = 0.0071
Sarcosine	84.22 (70.51; 122.7)	65.09 (30.28; 73.45)	92.13 (67.95; 113.4)	*p* = 0.0570
Alanine	15,841 (14,823; 17,128)	17,909 (16,745; 18,883)	**15,411 (14,137; 15,535)** ^**##**^	**p** = 0.0045
Beta‐alanine	337.8 (309.0; 367.1)	345.2 (304.4; 385.7)	345.9 (292.0; 391.6)	*p* = 0.9896
Taurine	3934 (3028; 4870)	**7173 (5889; 8407)** ^**∗**^	**3555 (3406; 5616)** ^**#**^	**p** = 0.0031
Renal cortex (μmol/kg)				
Betaine	1624 (1271; 2153)	1420 (1198; 1737)	1755 (1363; 2220)	*p* = 0.3738
Glycine	11,017 (10,528; 12,106)	9904 (9863; 10,719)	**9535 (8921; 10,185)** ^**∗∗**^	**p** = 0.0041
Sarcosine	11.03 (8.244; 13.10)	**6.038 (4.397; 7.851)** ^**∗**^	**11.69 (9.348; 13.07)** ^**#**^	**p** = 0.003
Alanine	19,261 (18,564; 21,443)	**14,509 (13,340; 15,693)** ^**∗**^	**13,800 (13,038; 15,875)** ^**∗∗**^	**p** = 0.0004
Beta‐alanine	168.0 (119.2; 219.9)	120.5 (92.52; 185.3)	107.1 (59.08; 126.6)	*p* = 0.0514
Taurine	7149 (6448; 8416)	**13,714 (11,214; 15,020)** ^**∗**^	**5805 (5110; 6706)** ^**##**^	**p** = 0.0001
Renal medulla (μmol/kg)				
Betaine	1862 (1782; 2434)	1571 (1454; 1824)	1850 (1305; 3120)	*p* = 0.2215
Glycine	10,215 (9256; 11,061)	9883 (9472; 10,079)	9580 (9265; 10,596)	*p* = 0.7020
Sarcosine	13.13 (11.56; 16.89)	**9.249 (8.423; 9.921)** ^**∗**^	**15.15 (14.73; 16.17)** ^**##**^	**p** = 0.0007
Alanine	14,120 (12,719; 15,130)	11,508 (10,987; 11,998)	**10,745 (10,010; 11,254)** ^**∗∗∗**^	**p** < 0.0001
Beta‐alanine	122.2 (104.5; 163.0)	122.7 (97.72; 154.8)	109.5 (65.78; 149.1)	*p* = 0.6343
Taurine	6806 (5405; 8472)	11,701 (7498; 13,072)	**5042 (4172; 7802)** ^**#**^	**p** = 0.022

*Note:* All data are expressed as the median (Q1; Q3) Kruskal–Wallis test followed by post hoc Dunn’s test. Bold values represent *p* values that are statistically significant.

^∗^ *p* < 0.05 vs. control.

^∗∗^ *p* < 0.01 vs. control.

^∗∗∗^ *p* < 0.001 vs. control.

^#^ *p* < 0.05 vs. spontaneously hypertensive.

^##^ *p* < 0.01 vs. spontaneously hypertensive.

## 4. Discussion

**Figure 1:**
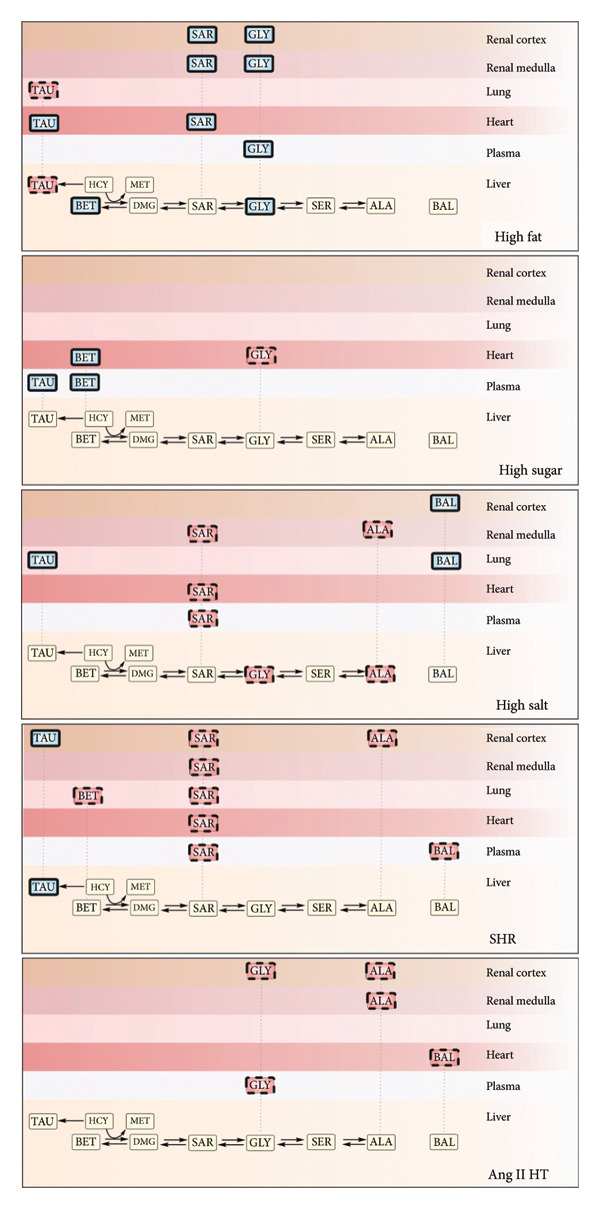
Influence of diet and hypertension on the metabolism of organic solutes. Intake of high fat, sugar, salt, spontaneous, and angiotensin II–induced hypertension increased (solid‐bold line) or decreased (dashed line) the levels of organic solutes in the plasma and tissues. Other metabolites (solid line) were unchanged or undetected. ALA; alanine, Ang II HT; angiotensin II–induced hypertension, BAL; beta‐alanine, BET; betaine, DMG; dimethylglycine, GLY; glycine, HCY; homocysteine, MET; methionine, PYR; pyruvate, SAR; sarcosine, SER; serine, SHR; spontaneously hypertensive rat, TAU; taurine.

The current study explores the impact of diet and hypertension on the levels of glycine and other metabolic‐ or structural‐related organic solutes in the plasma and tissues of rats. High‐fat diet with caloric restriction increased the tissue levels of methylglycines (sarcosine, betaine) and glycine. In contrast, high salt intake and hypertension reduced the concentrations of methyl/glycine species, while favoring the accumulation of taurine in the tissues. Both excessive salt and hypertension models lowered alanine levels in the kidneys. Beta‐alanine was higher in the kidney and lung of rats on high salt, but lower in the cardiovascular system of hypertensive models. High‐sugar diet with caloric restriction increased circulating levels of betaine and taurine, but its impact on tissue levels of organic solutes was less pronounced (Figure 1). Below, we discuss in detail the metabolic changes induced by diet and hypertension.

### 4.1. Sarcosine and Glycine

Sarcosine is an intermediate product in the reversible demethylation of glycine. We found that the plasmatic, renal, and cardiac levels of sarcosine were lower in SHRs and rats on high salt, but not affected in Ang II hypertensive rats and rats on high‐sugar diet. These data suggest that the alternation of sarcosine may be related with sodium handling independently of angiotensin activity. Previous reports showed that methylation can alter the activity of sodium transporters that are crucial for the regulation of salt reabsorption in the kidneys [7]. Incubation of cells with sarcosine increased demethylation enzymes, methyl donors, and methylation potential of cells. Importantly, decreased systemic levels of sarcosine were reported in diabetic patients, while decreasing circulating sarcosine was associated with aging or dietary restrictions [14, 15]. Furthermore, sarcosine levels negatively correlated with the severity of heart failure in patients [16]. Thus, a decrease in systemic sarcosine is associated with pathological conditions that may arise following high salt intake and hypertension.

Similarly, decreased levels of glycine in the organism were related to higher incidence of myocardial infarction and Type 2 diabetes [17]. In our study, the levels of glycine were reduced in the heart of rats on high‐sugar diet. This alternation may result from the activation of glycolysis due to higher availability of sugar in heart that lowers the demand for oxidation of amino acids, including glycine. Additionally, we determined lower glycine levels in the plasma and renal cortex of Ang II hypertensive rats, but systemic glycine was unaltered in SHRs. Previous studies have reported decreased glycine in the urine and kidneys of hypertensive rats [18, 19]. In patients, higher glycine levels have been associated with a lower risk to develop hypertension [20]. In addition, it was reported that reduced glycine levels may decrease renal blood flow and stimulate renin production in the kidneys, thus promoting the vasoconstriction of Ang II [21]. Furthermore, methylation can negatively regulate the expression of angiotensinogen [22]. Hence, we anticipate that there might be a cross‐talk between glycine metabolism and Ang II signaling.

The content of glycine in the high‐fat diet was approximately 5 times higher than that of the control diet [10]. Consequently, we observed accumulation of plasmatic, hepatic, and renal glycine together with higher cardiac and renal sarcosine. In contrast, clinical studies have shown decreased plasma glycine concentrations in obese patients, suggesting that diet composition is critical determinant of metabolic outcomes [23–25]. Similarly, serum glycine was lower in rats fed a high‐fat diet, but increased when the high‐fat diet was deficient on various amino acids [26]. Both glycine and sarcosine are components of one‐carbon metabolism that control the excess of S‐adenosylmethionine (SAM), a donor of methyl groups. Plasmatic levels of SAM positively correlate with fat mass and adiposity, probably to regulate lipid metabolism by methylation processes [6]. Additionally, glycine is essential for the conjugation of bile acids in the liver thus increasing their solubility and transport to the gut. Previously, we showed that high‐fat diet with caloric restriction was associated with the development of steatohepatitis and dyslipidemia [10]. Therefore, we posit that the accumulation of glycine in the liver is related to the stimulated lipid metabolism by high‐fat diet. These tissue‐specific metabolic alterations should be considered in future studies investigating the impact of high‐fat diet on metabolism.

### 4.2. Betaine and Taurine

The transfer of methyl group from betaine to homocysteine results in the formation of methionine, whereby betaine is converted to dimethylglycine. Subsequent demethylation of dimethylglycine results in the formation of sarcosine. Alternatively, homocysteine enters the transsulfuration pathway to produce various sulfur derivatives including taurine [27]. In our study, high‐fat diet increased the levels of betaine in the liver of rats. Several reports confirmed the protective role of betaine against high‐fat diet‐induced hepatic steatosis [28]. Betaine protected the liver from the accumulation of lipids and homocysteine induced by high‐fat diet [28] and reversed liver injury by restoring the methylation index [29]. In contrast, we determined lower lung betaine levels in SHRs. Previously, we showed that tissue betaine levels in older hypertensive rats were lower not only in the lungs but also in plasma, liver, and renal medulla [30]. These data suggest that betaine concentration decreases with the development of hypertension and may contribute to poor methylation status in the tissues.

Next, we found lower levels of taurine in the liver of rats on high‐fat diet. This may be due to the lower cystine and methionine content in the high‐fat diet compared to the control diet [10], reducing the availability of these taurine precursors. Taurine is together with glycine essential for bile acid conjugation in the liver. Therefore, lower taurine may contribute to lipid accumulation and the development of hepatic steatosis. As a result, decrease in hepatic taurine may increase glycine demand to provide the formation of bile salts that are crucial for digestion and absorption, particularly under high‐fat intake, as observed in our study. Additionally, we determined lower lung levels of taurine, but higher cardiac taurine due to high‐fat intake. We suppose that high‐fat diet may induce the translocation of taurine from liver and lung to the heart to provide cardioprotection. The high levels of taurine in the heart support contractile function, osmotic balance, antioxidant activity, and stabilize membranes [31].

Otherwise, high salt intake resulted in the accumulation of taurine in the lungs. It was shown that high salt increased sodium and water content in the lungs of rats on high salt [32], suggesting that the increase in taurine may serve as osmoprotection against stress induced in the lung by excessive salt intake. In hypertensive rats, taurine was higher in the liver and renal cortex. Similar levels of renal taurine were reported by Dawson et al. in stroke‐prone hypertensive rats [33]. Conversely, decreased taurine concentrations in the urine and heart have been previously reported in hypertensive rats [18]. It was reported that taurine prevents the development of hypertension by promoting the release of renal kallikrein, a key regulator of sodium homeostasis [34]. We hypothesize that taurine accumulation may help mitigate stress conditions in the liver and kidney associated with hypertension [35–37].

High‐sugar diet increased the levels of circulating betaine and taurine. Betaine was also higher in the heart of rats on high‐sugar diet. Increased plasmatic glucose due to high‐sugar diet alters osmotic pressure. Since betaine and taurine are major osmolytes, they probably regulate osmotic changes in plasma induced by glucose. However, high fructose exposure did not alter taurine plasma levels in Chinese residents, suggesting that the clinical relevance of these findings needs to be further examined [38]. Surprisingly, high salt diet did not alter the levels of betaine in the tissues of rats, suggesting that betaine is not a dominant regulator of sodium handling in the tissues.

### 4.3. Alanine and Beta‐Alanine

Although alanine and beta‐alanine are structurally related, they do not share a direct metabolic connection. Interestingly, we found that the two models of hypertension shared only one pattern, namely, the downregulation of alanine in the kidneys and beta‐alanine in the cardiovascular system. Similarly, lower plasmatic levels of alanine were found in older hypertensive rats [39]. In line, higher urinary excretion of alanine was related to the development of hypertension [40]. However, others showed increase in cardiac and renal alanine together with serum beta‐alanine in hypertensive rats [19]. We further revealed that high salt intake decreased alanine levels in the renal medulla of rats. Previous reports showed that high salt intake and hypertension lower the expression of sodium‐dependent transporters for alanine in the renal cortex [41]. Interestingly, the decrease was more pronounced in salt‐sensitive than salt‐resistant rats [42]. Since reduced sodium–alanine cotransport is associated with alternations in renal salt and fluid handling, it may contribute to the development of salt sensitivity and hypertension.

Beta‐alanine is a substrate for carnosine, a cardioprotective agent [43]. Therefore, we speculate that lower beta‐alanine levels in hypertensive rats may be related to reduce cardiac defense of carnosine. In contrast, high salt intake resulted in the accumulation of beta‐alanine in the lung and renal cortex of rats. Beta‐alanine is together with taurine a major substrate for beta‐amino acid transporters that regulate osmoregulation by maintaining sodium gradient across the membrane [44]. Therefore, an increase of beta‐alanine and taurine in the lung may be a protective response to osmotic stress induced by high salt.

Next, high salt intake decreased alanine in the liver of rats. Alanine is in the liver converted to glucose and urea [45]. It was reported that high salt intake altered glucose metabolism in the liver and glucose reabsorption in the kidneys [46, 47]. In addition, high salt intake promoted hepatic urea production and renal urea recycling to maintain the osmotic balance [48]. These data suggest that excessive salt may induce alanine metabolism in the liver to promote gluconeogenesis and the activity of the urea cycle. Interestingly, the changes in hepatic alanine levels mediated by high salt correlated with the changes in hepatic glycine levels. Similarly, Ang II hypertensive rats showed reduced glycine and alanine in the kidney, while high salt intake and spontaneous hypertension reduced alanine together with sarcosine in the kidneys. Reduced alanine levels may be related to higher pyruvate utilization, thus affecting the availability of intermediates involved in glycine/sarcosine pathway. Correlations between alanine and glycine were observed under fasting conditions or in diabetic patients [49, 50]. In contrast, a negative correlation between alanine and glycine was observed in patients with metabolic syndrome [51]. These structurally related amino acids were also shown to provide cytoprotection in the renal cells and liver [52]. Interestingly, despite the higher alanine content in the high‐fat diet, no changes were observed in the plasma or tissues of the rats. This contrasts with studies in obese patients that have reported increased plasma alanine levels [25]. In addition, a high‐fat diet deficient in various amino acids either increased or decreased plasma levels of alanine, highlighting the importance of diet composition in determining the systemic levels of amino acids [26].

## 5. Limitations

We acknowledge that the study was limited to descriptive metabolic analysis and did not include interventional experiments. Future research should focus on functional approaches, such as supplementation or inhibition of selected amino acids, to determine whether modulating these metabolites can ameliorate hypertension or cardiovascular dysfunction. Such studies will be essential to establish causal relationships and evaluate the therapeutic potential of amino acid‐targeted interventions.

## 6. Conclusions

Our findings indicate that diet and hypertension significantly alter the levels of amino acids and nutrients that supply the one‐carbon metabolism. We found that high‐fat diet increased glycine and its methylated derivatives, while high salt intake and hypertension decreased these species in the organism. A high‐sugar diet increased plasmatic betaine and taurine, but the impact on tissue metabolism was minor. To sum up, the changes in organic solutes did not follow a consistent pattern across experimental conditions, reflecting the complexity of metabolic responses to unbalanced diet and hypertension.

## Disclosure

All authors read and approved the final manuscript and agreed to be accountable for all aspects of the work in ensuring that questions related to the accuracy or integrity of any part of the work are appropriately investigated and resolved.

## Conflicts of Interest

The authors declare no conflicts of interest.

## Author Contributions

All authors contributed to the study conception and design. Kristina Smoradkova, Mateusz Szudzik, Klaudia Maksymiuk, and Emilia Samborowska performed material preparation, data collection, and analysis. Lenka Tomasova interpreted the data and wrote the manuscript. Adrian Drapala and Marcin Ufnal interpreted the data and revised the manuscript.

## Funding

Lenka Tomasova was funded by the EU NextGeneration through the Recovery and Resilience Plan for Slovakia under the Project No. 09I03‐03‐V04‐00468/2024/VA and the VEGA Grant Agency of the Slovak Republic under the Project No. 2/0066/23.

## Data Availability

The data that support the findings of this study are available from the corresponding author upon reasonable request.

## References

[1] Ludwig D. S. , Willett W. C. , Volek J. S. , and Neuhouser M. L. , Dietary Fat: From Foe to Friend?, Science (New York, NY). (2018) 362, no. 6416, 764–770, 10.1126/science.aau2096, 2-s2.0-85056581871. 30442800

[2] Kearney P. M. , Whelton M. , Reynolds K. , Muntner P. , Whelton P. K. , and He J. , Global Burden of Hypertension: Analysis of Worldwide Data, Lancet (London, England). (2005) 365, no. 9455, 217–223, 10.1016/s0140-6736(05)17741-1. 15652604

[3] Elliott J. , Elliott J. , Syme H. M. , and Jepson R. E. , Physiology of Blood Pressure Regulation and Pathophysiology of Hypertension, Hypertension in the Dog and Cat, 2020, Springer International Publishing, Cham, 3–30, 10.1007/978-3-030-33020-0_1.

[4] Locasale J. W. , Serine, Glycine and One-Carbon Units: Cancer Metabolism in Full Circle, Nature Reviews Cancer. (2013) 13, no. 8, 572–583, 10.1038/nrc3557, 2-s2.0-84881177291. 23822983 PMC3806315

[5] Hong X. , Miao K. , Cao W. et al., Association Between DNA Methylation and Blood Pressure: A 5-Year Longitudinal Twin Study, Hypertension. (2023) 80, no. 1, 169–181, 10.1161/HYPERTENSIONAHA.122.19953. 36345830

[6] Amany K. E. , Giel N. , Maria V.-G. et al., S-Adenosylmethionine is Associated With Fat Mass and Truncal Adiposity in Older Adults, The Journal of Nutrition. (2013) 143, no. 12, 1982–1988, 10.3945/jn.113.179192, 2-s2.0-84886949032. 24068793

[7] Yan Y. , Liu H. , Abedini A. et al., Unraveling the Epigenetic Code: Human Kidney DNA Methylation and Chromatin Dynamics in Renal Disease Development, Nature Communications. (2024) 15, no. 1, 10.1038/s41467-024-45295-Y. PMC10824731 38287030

[8] Liu N. , Chen Y. , Wang Y. et al., The Underlying Mechanisms of DNA Methylation in High Salt Memory in Hypertensive Vascular Disease, Scientific Reports. (2024) 14, no. 1, 10.1038/s41598-024-51279-1. PMC10776617 38195688

[9] Gomez-Alonso MdC. , Kretschmer A. , Wilson R. et al., DNA Methylation and Lipid Metabolism: An Ewas of 226 Metabolic Measures, Clinical Epigenetics. (2021) 13, no. 1, 10.1186/s13148-020-00957-8. PMC7789600 33413638

[10] Szudzik M. , Hutsch T. , Chabowski D. , Zajdel M. , and Ufnal M. , Normal Caloric Intake With High-Fat Diet Induces Metabolic Dysfunction-Associated Steatotic Liver Disease and Dyslipidemia Without Obesity in Rats, Scientific Reports. (2024) 14, no. 1, 10.1038/s41598-024-74193-Y. PMC11445425 39354056

[11] Gawryś-Kopczyńska M. , Szudzik M. , Samborowska E. et al., Spontaneously Hypertensive Rats Exhibit Increased Liver Flavin Monooxygenase Expression and Elevated Plasma Tmao Levels Compared to Normotensive and Ang II-Dependent Hypertensive Rats, Frontiers in Physiology. (2024) 15, 10.3389/fphys.2024.1340166. PMC11046708 38681141

[12] Tomasova L. , Maksymiuk K. , Chabowski D. , Samborowska E. , and Ufnal M. , Mice, Rats and Guinea Pigs Exhibit Significant Variations in the Plasma, Urine and Tissue Levels of Taurine, Betaine, Sarcosine and Other Osmolyte-Active Amino Acids, Discovery Medicine. (2023) 35, no. 177, 492–502, 10.24976/Discov.Med.202335177.50. 37553303

[13] Maksymiuk K. M. , Szudzik M. , Samborowska E. , Chabowski D. , Konop M. , and Ufnal M. , Mice, Rats, and Guinea Pigs Differ in Fmos Expression and Tissue Concentration of Tmao, a Gut Bacteria-Derived Biomarker of Cardiovascular and Metabolic Diseases, Plos One. (2024) 19, no. 1, 10.1371/journal.pone.0297474. PMC10807837 38266015

[14] Calvani R. , Rodriguez-Mañas L. , Picca A. et al., Identification of a Circulating Amino Acid Signature in Frail Older Persons With Type 2 Diabetes Mellitus: Results From the Metabofrail Study, Nutrients. (2020) 12, no. 1, 10.3390/nu12010199. PMC7019630 31940925

[15] Walters R. O. , Arias E. , Diaz A. et al., Sarcosine is Uniquely Modulated by Aging and Dietary Restriction in Rodents and Humans, Cell Reports. (2018) 25, no. 3, 663–676.e666, 10.1016/j.celrep.2018.09.065, 2-s2.0-85054742744. 30332646 PMC6280974

[16] Yang C. , Shi Z. , Bao L. , Xv X. , Jiang D. , and You L. , Targeted Metabolomic Analysis of Serum Amino Acids in Heart Failure Patients, Amino Acids. (2024) 56, no. 1, 10.1007/s00726-024-03385-7. PMC10940394 38483649

[17] Wittemans L. B. L. , Lotta L. A. , Oliver-Williams C. et al., Assessing the Causal Association of Glycine With Risk of Cardio-Metabolic Diseases, Nature Communications. (2019) 10, no. 1, 10.1038/s41467-019-08936-1, 2-s2.0-85062584534. PMC6400990 30837465

[18] Akira K. , Masu S. , Imachi M. , Mitome H. , and Hashimoto T. , A Metabonomic Study of Biochemical Changes Characteristic of Genetically Hypertensive Rats Based on 1H NMR Spectroscopic Urinalysis, Hypertension Research. (2012) 35, no. 4, 404–412, 10.1038/hr.2011.182, 2-s2.0-84859597915. 22089538

[19] Li Y. , Xie D. , Li L. , and Jiang P. , Comprehensive Analysis of Metabolic Changes in Spontaneously Hypertensive Rats, Clinical and Experimental Hypertension. (2023) 45, no. 1, 10.1080/10641963.2023.2190529. 36922753

[20] Dietrich S. , Floegel A. , Weikert C. et al., Identification of Serum Metabolites Associated With Incident Hypertension in the European Prospective Investigation Into Cancer and Nutrition-Potsdam Study, Hypertension. (2016) 68, no. 2, 471–477, 10.1161/hypertensionaha.116.07292, 2-s2.0-84973334448. 27245178

[21] Thomsen K. , Nielsen C. B. , and Flyvbjerg A. , Effects of Glycine on Glomerular Filtration Rate and Segmental Tubular Handling of Sodium in Conscious Rats, Clinical and Experimental Pharmacology and Physiology. (2002) 29, no. 5-6, 449–454, 10.1046/j.1440-1681.2002.03683.x, 2-s2.0-0036096177. 12010191

[22] Demura M. , Demura Y. , Takeda Y. , and Saijoh K. , Dynamic Regulation of the Angiotensinogen Gene by DNA Methylation, Which is Influenced by Various Stimuli Experienced in Daily Life, Hypertension Research. (2015) 38, no. 8, 519–527, 10.1038/hr.2015.42, 2-s2.0-84938807850. 25809578

[23] Takashina C. , Tsujino I. , Watanabe T. et al., Associations Among the Plasma Amino Acid Profile, Obesity, and Glucose Metabolism in Japanese Adults With Normal Glucose Tolerance, Nutrition and Metabolism. (2016) 13, no. 1, 10.1186/s12986-015-0059-5, 2-s2.0-84955236667. PMC4717594 26788116

[24] Labonte C. C. , Farsijani S. , Marliss E. B. et al., Plasma Amino Acids Vs Conventional Predictors of Insulin Resistance Measured by the Hyperinsulinemic Clamp, Journal of the Endocrine Society. (2017) 1, no. 7, 861–873, 10.1210/js.2016-1108. 29264537 PMC5686697

[25] Tan H. C. , Hsu J. W. , Tai E. S. , Chacko S. , Kovalik J. P. , and Jahoor F. , The Impact of Obesity-Associated Glycine Deficiency on the Elimination of Endogenous and Exogenous Metabolites via the Glycine Conjugation Pathway, Frontiers in Endocrinology. (2024) 15, 10.3389/fendo.2024.1343738. PMC11023637 38633754

[26] Nishi H. , Goda Y. , Okino R. et al., Metabolic Effects of Short-Term High-Fat Intake Vary Depending on Dietary Amino Acid Composition, Current Developments in Nutrition. (2024) 8, no. 6, 10.1016/j.cdnut.2024.103768. PMC11208941 38939648

[27] Obeid R. , The Metabolic Burden of Methyl Donor Deficiency With Focus on the Betaine Homocysteine Methyltransferase Pathway, Nutrients. (2013) 5, no. 9, 3481–3495, 10.3390/nu5093481, 2-s2.0-84884175034. 24022817 PMC3798916

[28] Arumugam M. K. , Paal M. C. , Donohue T. M. , Ganesan M. , Osna N. A. , and Kharbanda K. K. , Beneficial Effects of Betaine: A Comprehensive Review, Biology. (2021) 10, no. 6, 10.3390/biology10060456. PMC8224793 34067313

[29] Kharbanda K. K. , Mailliard M. E. , Baldwin C. R. , Beckenhauer H. C. , Sorrell M. F. , and Tuma D. J. , Betaine Attenuates Alcoholic Steatosis by Restoring Phosphatidylcholine Generation via the Phosphatidylethanolamine Methyltransferase Pathway, Journal of Hepatology. (2007) 46, no. 2, 314–321, 10.1016/j.jhep.2006.08.024, 2-s2.0-33845676630. 17156888

[30] Mogilnicka I. , Jaworska K. , Koper M. et al., Hypertensive Rats Show Increased Renal Excretion and Decreased Tissue Concentrations of Glycine Betaine, a Protective Osmolyte With Diuretic Properties, Plos One. (2024) 19, no. 1, 10.1371/journal.pone.0294926. PMC10760924 38166023

[31] Schaffer S. W. , Ju Jong C. , Kc R. , and Azuma J. , Physiological Roles of Taurine in Heart and Muscle, Journal of Biomedical Science. (2010) 17, no. 1, 10.1186/1423-0127-17-S1-S2, 2-s2.0-77956022823. PMC2994395 20804594

[32] Rossitto G. , Mary S. , Chen J. Y. et al., Tissue Sodium Excess is Not Hypertonic and Reflects Extracellular Volume Expansion, Nature Communications. (2020) 11, no. 1, 10.1038/s41467-020-17820-2. PMC7445299 32839436

[33] Dawson R.Jr., Liu S. , Jung B. , Messina S. , and Eppler B. , Effects of High Salt Diets and Taurine on the Development of Hypertension in the Stroke-Prone Spontaneously Hypertensive Rat, Amino Acids. (2000) 19, no. 3-4, 643–665, 10.1007/s007260070014. 11140367

[34] Ideishi M. , Miura S.-I , Sakai T. , Sasaguri M. , Misumi Y. , and Arakawa K. , Taurine Amplifies Renal Kallikrein and Prevents Salt-Induced Hypertension in Dahl Rats, Journal of Hypertension. (1994) 12, no. 6, 653–662, 10.1097/00004872-199406000-00005. 7963490

[35] Szlęzak D. , Bronowicka-Adamska P. , Hutsch T. , Ufnal M. , and Wróbel M. , Hypertension and Aging Affect Liver Sulfur Metabolism in Rats, Cells. (2021) 10, no. 5, 10.3390/cells10051238. PMC8157544 34069923

[36] Koga Y. , Hirooka Y. , Araki S. , Nozoe M. , Kishi T. , and Sunagawa K. , High Salt Intake Enhances Blood Pressure Increase During Development of Hypertension via Oxidative Stress in Rostral Ventrolateral Medulla of Spontaneously Hypertensive Rats, Hypertension Research. (2008) 31, no. 11, 2075–2083, 10.1291/hypres.31.2075, 2-s2.0-58849118857. 19098380

[37] Khraibi A. A. and Knox F. G. , Effect of Acute Renal Decapsulation on Pressure Natriuresis in SHR and WKY Rats, American Journal of Physiology. (1989) 257, no. 5 Pt 2, F785–F789, 10.1152/ajprenal.1989.257.5.F785. 2589482

[38] Rang O. , Qin X. , Tang Y. et al., The Effect of Fructose Exposure on Amino Acid Metabolism Among Chinese Community Residents and Its Possible Multi-Omics Mechanisms, Scientific Reports. (2023) 13, no. 1, 10.1038/s41598-023-50069-5. PMC10733306 38123624

[39] Kondziella D. , Zetterberg H. , Haugen E. , and Fu M. , Hypertension in Spontaneously Hypertensive Rats Occurs Despite Low Plasma Levels of Homocysteine, Physiological Research. (2008) 57, no. 3, 487–490, 10.33549/physiolres.931181. 17298201

[40] Mahbub M. H. , Yamaguchi N. , Takahashi H. et al., Association of Plasma Free Amino Acids With Hyperuricemia in Relation to Diabetes Mellitus, Dyslipidemia, Hypertension and Metabolic Syndrome, Scientific Reports. (2017) 7, no. 1, 10.1038/s41598-017-17710-6, 2-s2.0-85038232681. PMC5732272 29247200

[41] Pinto V. , Pinho M. J. , and Soares-Da-Silva P. , Renal Amino Acid Transport Systems and Essential Hypertension, The FaseB Journal. (2013) 27, no. 8, 2927–2938, 10.1096/fj.12-224998, 2-s2.0-84881192710. 23616567

[42] Li L. , Zhong S. J. , Hu S. Y. , Cheng B. , Qiu H. , and Hu Z. X. , Changes of Gut Microbiome Composition and Metabolites Associated With Hypertensive Heart Failure Rats, BMC Microbiology. (2021) 21, no. 1, 10.1186/s12866-021-02202-5. PMC8097775 33952214

[43] Creighton J. V. , de Souza Gonçalves L. , Artioli G. G. et al., Physiological Roles of Carnosine in Myocardial Function and Health, Advances in Nutrition. (2022) 13, no. 5, 1914–1929, 10.1093/advances/nmac059. 35689661 PMC9526863

[44] Hammer M. A. and Baltz J. M. , Beta-Alanine but Not Taurine Can Function as an Organic Osmolyte in Preimplantation Mouse Embryos Cultured From Fertilized Eggs, Molecular Reproduction and Development. (2003) 66, no. 2, 153–161, 10.1002/mrd.10343, 2-s2.0-0042355346. 12950102

[45] Dixon G. , Nolan J. , Mcclenaghan N. , Flatt P. R. , and Newsholme P. , A Comparative Study of Amino Acid Consumption by Rat Islet Cells and the Clonal Beta-Cell Line Brin-BD11: The Functional Significance of L-Alanine, Journal of Endocrinology. (2003) 179, no. 3, 447–454, 10.1677/joe.0.1790447, 2-s2.0-0347287092. 14656214

[46] Zhao Y. , Gao P. , Sun F. et al., Sodium Intake Regulates Glucose Homeostasis Through the Pparδ/Adiponectin-Mediated Sglt2 Pathway, Cell Metabolism. (2016) 23, no. 4, 699–711, 10.1016/j.cmet.2016.02.019, 2-s2.0-84963706055. 27053360

[47] Wu Q. , Burley G. , Li L. C. , Lin S. , and Shi Y. C. , The Role of Dietary Salt in Metabolism and Energy Balance: Insights Beyond Cardiovascular Disease, Diabetes, Obesity and Metabolism. (2023) 25, no. 5, 1147–1161, 10.1111/dom.14980. PMC10946535 36655379

[48] Kitada K. , Daub S. , Zhang Y. et al., High Salt Intake Reprioritizes Osmolyte and Energy Metabolism for Body Fluid Conservation, Journal of Clinical Investigation. (2017) 127, no. 5, 1944–1959, 10.1172/jci88532, 2-s2.0-85018953498. 28414295 PMC5409074

[49] Robert J. J. , Beaufrere B. , Koziet J. et al., Whole Body De Novo Amino Acid Synthesis in Type I (Insulin-Dependent) Diabetes Studied With Stable Isotope-Labeled Leucine, Alanine, and Glycine, Diabetes. (1985) 34, no. 1, 67–73, 10.2337/diab.34.1.67, 2-s2.0-0021970330. 3880550

[50] Ye J. , Gu Y. , Zhang F. et al., IDH1 Deficiency Attenuates Gluconeogenesis in Mouse Liver by Impairing Amino Acid Utilization, Proceedings of the National Academy of Sciences of the United States of America. (2017) 114, no. 2, 292–297, 10.1073/pnas.1618605114, 2-s2.0-85009259942. 28011762 PMC5240724

[51] Kamaura M. , Nishijima K. , Takahashi M. , Ando T. , Mizushima S. , and Tochikubo O. , Lifestyle Modification in Metabolic Syndrome and Associated Changes in Plasma Amino Acid Profiles, Circulation Journal: Official Journal of the Japanese Circulation Society. (2010) 74, no. 11, 2434–2440, 10.1253/circj.cj-10-0150, 2-s2.0-78349286984. 20834187

[52] Van den Eynden J. , Ali S. S. , Horwood N. et al., Glycine and Glycine Receptor Signalling in Non-Neuronal Cells, Frontiers in Molecular Neuroscience. (2009) 2, 10.3389/neuro.02.009.2009, 2-s2.0-84858265031. PMC2737430 19738917

